# Protective Role of Dietary Berries in Cancer

**DOI:** 10.3390/antiox5040037

**Published:** 2016-10-19

**Authors:** Aleksandra S. Kristo, Dorothy Klimis-Zacas, Angelos K. Sikalidis

**Affiliations:** 1 Department of Nutrition and Dietetics, Istanbul Yeni Yuzyil University, Yilanli Ayasma Caddesi No. 26, Istanbul 34010, Turkey; as545@cornell.edu; 2 Food Science and Human Nutrition, University of Maine, Orono, ME 04469, USA; Dorothy_Klimis_Zacas@umit.maine.edu

**Keywords:** antioxidants, anthocyanins, cancer, chemoprevention, edible berries, flavonoids, phytochemicals

## Abstract

Dietary patterns, including regular consumption of particular foods such as berries as well as bioactive compounds, may confer specific molecular and cellular protection in addition to the overall epidemiologically observed benefits of plant food consumption (lower rates of obesity and chronic disease risk), further enhancing health. Mounting evidence reports a variety of health benefits of berry fruits that are usually attributed to their non-nutritive bioactive compounds, mainly phenolic substances such as flavonoids or anthocyanins. Although it is still unclear which particular constituents are responsible for the extended health benefits, it appears that whole berry consumption generally confers some anti-oxidant and anti-inflammatory protection to humans and animals. With regards to cancer, studies have reported beneficial effects of berries or their constituents including attenuation of inflammation, inhibition of angiogenesis, protection from DNA damage, as well as effects on apoptosis or proliferation rates of malignant cells. Berries extend effects on the proliferation rates of both premalignant and malignant cells. Their effect on premalignant cells is important for their ability to cause premalignant lesions to regress both in animals and in humans. The present review focuses primarily on in vivo and human dietary studies of various berry fruits and discusses whether regular dietary intake of berries can prevent cancer initiation and delay progression in humans or ameliorate patients’ cancer status.

## 1. Background

Cancer, the uncontrolled growth of cells which can invade and spread to distant sites of the body, is a global health problem with high mortality and disability rates. The most common forms of cancer in males are: lung, prostate, colorectal, stomach, and liver cancer; whereas in females: breast, colorectal, lung, uterine/cervix, and stomach cancer. Prevention, in coordination with monitoring leading to early and accurate diagnosis when a case is confirmed, is critical, since most therapeutic options do not confer cure but rather a deceleration of cancer progression aiming at life extension and improvement of patients’ life quality, albeit with serious and often debilitating side-effects. According to the latest available World Health Organization (WHO) global report (World Cancer Report 2014, as updated in 2015) [1] cancer is one of the leading causes of morbidity and mortality worldwide, reaching approximately 14 million new cases and 8.2 million cancer related deaths in 2012 [1] (Table 1). While new cancer incidence is expected to rise by 70% by the year 2034, approximately 35% of cancer deaths are attributed to the five leading behavioral and dietary risks: high body mass index (BMI), low fruit and vegetable intake, lack of physical activity, tobacco use (primarily smoking) and harmful alcohol use [1].

According to the latest published report by the Center for Disease Control (CDC), in the US alone 1,685,210 new cases of cancer are estimated in 2016 of which 841,390 pertain to males and 843,820 to females. The same report projects an estimated 595,690 deaths from cancer in 2016 in the entire US, of which 314,290 refer to males and 281,400 to females [2]. According to the Agency for Healthcare Research and Quality (AHRQ) in the USA (all 50 States) the direct medical costs (total of all health care costs) for cancer in 2011 reached US $88.7 billion [2,3,4]. In its latest global report on cancer issued in 2014 as updated in 2015 [1], WHO stresses that being overweight or obese on one hand and consuming unhealthy diets with low fruit and vegetable intakes on the other, constitute important, yet modifiable risk factors which significantly increase cancer risk for both genders. A plethora of in vitro, in vivo as well as human studies has suggested diet as a critical factor for reducing cancer risk with a particular emphasis on certain foods/food groups, to which specific potential for cancer risk reduction has been attributed [1]. Diets rich in fruits and vegetables have been associated with a reduced risk of cancer [5,6,7]. Many non-nutrient plant compounds known as phytochemicals, including numerous phenolic compounds, have been identified, and found to exert anti-cancer activity [5,8]. In this context, there has been an increasing interest in the role of polyphenols and other phytochemicals in cancer, particularly for their anti-oxidant and anti-inflammatory properties. The intake of bioactive compounds such as flavonoids has been inversely correlated with systemic inflammatory markers in human populations [9,10]. Protective role of certain foods against cancer, while clearly suggested, cannot easily be delineated as food items are highly complex, whereas protective compounds naturally found in foods, arguably act synergistically when present in an optimal balance. Therefore, research on the effects of whole food consumption and/or dietary schemes in relation to cancer risk is being increasingly conducted as deemed more physiologically and practically significant.

## 2. Cancer Development and Associated Mechanisms

**Figure 1:**
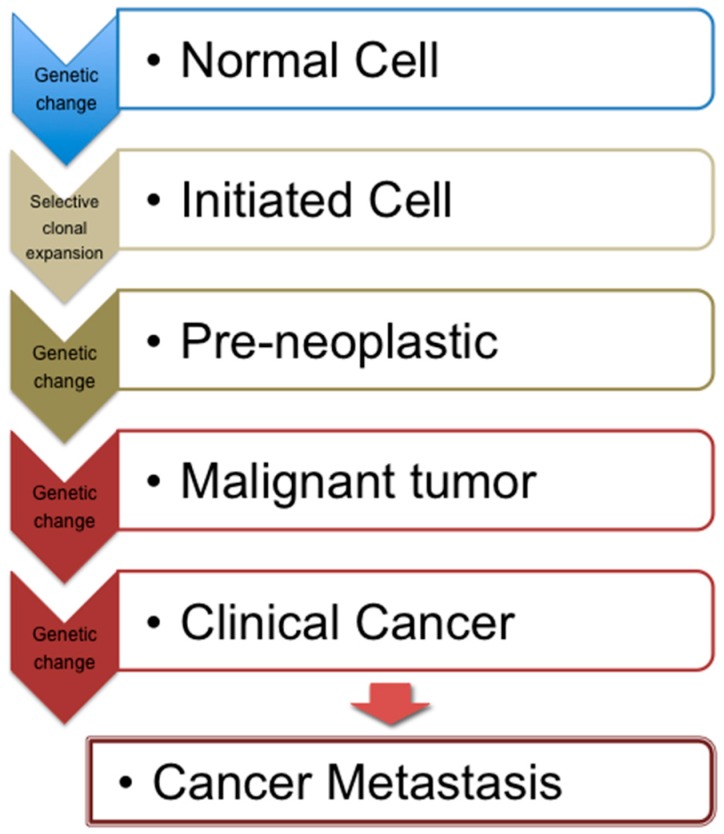
Schematic depiction of cancer development/progression.

Cancer can be viewed as a gradual generation and development of a tumor, which from a functional perspective can be divided into three phases: initiation, promotion and progression. Genomic changes such as point mutations, gene deletion and amplification and chromosomal rearrangements, all mark initiation, subsequently committing the cell to an irreversible status. In order for a tumor to be formed however, survival and clonal expansion of these initiated cells is required (Figure 1). Growth of tumor and metastasis (if it occurs) are characteristics of the progression phase. Development of cancer is a multistage process involving multiple genetic and epigenetic events occurring at varying rates. In order for cancer to develop, the acquisition of all six properties below is required: (i) self-sufficient proliferation; (ii) insensitivity to anti-proliferative signals; (iii) evasion of apoptosis/T-cell control; (iv) unlimited cellular replication ability; (v) maintenance of vascularization; and (vi) tissue invasion and metastasis. These alterations result from a combination of proto-oncogene activation, inactivation of tumor suppressor genes and inactivation of genomic stability genes.

**Figure 2:**
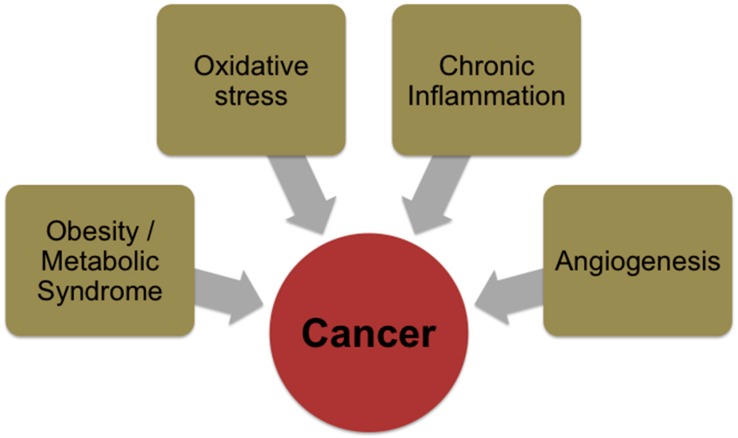
Major nutrition/diet-related factors/stressors that can contribute to cancer.

Several conditions and/or mechanisms that function as risk modulators for cancer including oxidative stress/chronic inflammation, obesity/metabolic syndrome, angiogenesis, apoptosis, autophagy and proliferation, have been proposed to explain cancer. Depending on the type/case of cancer any of the above and/or a combination may lead to cancer manifestation or progression (Figure 2). There is however no definitive mode of action via which cancer is initiated and hence prediction of cancer occurrence is not possible, while from a statistical standpoint cancer prevention is possible to a certain degree.

**Figure 3:**
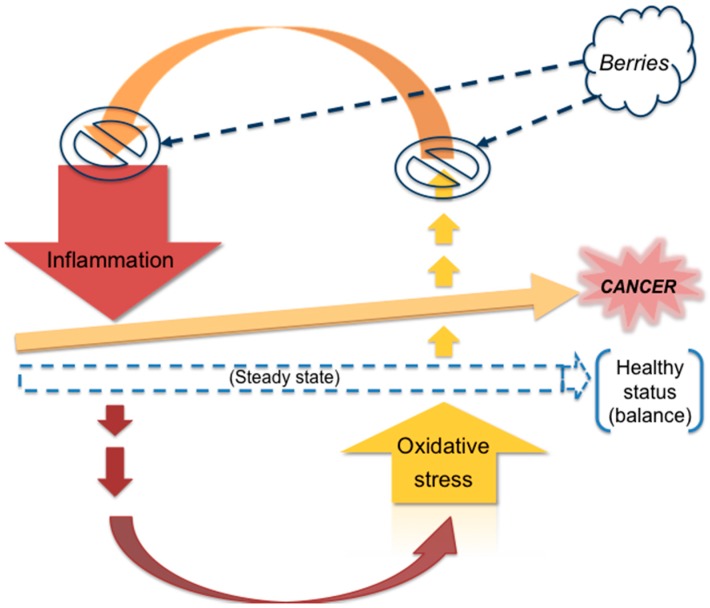
Conceptual schematic representation depicting the contribution of oxidative stress and inflammation in the deregulation/disturbance of homeostatic balance in a cell, thus leading to cancer. Berries by means of their numerous constituents (synergistic action) can interrupt this vicious cycle, thereby extending protective effects against cancer.

As cancer is a step-wise procedure, several factors may influence its initiation, development and progression. Oxidative stress can activate a variety of transcription factors including nuclear factor kappa B (NF-κB), activator protein-1 (AP-1), p53, hypoxia-inducible factor-1α (HIF-1α), peroxisome proliferator-activated receptor gamma (PPAR-γ or PPARG), β-catenin/Wnt, and NF-E2 related factor-2 (Nrf2). Activation of these transcription factors can lead to the expression of over 500 different genes, including those for growth factors, inflammatory cytokines, chemokines, cell cycle regulatory molecules, and anti-inflammatory molecules [11]. Chronic oxidative stress and inflammation can induce the events that essentially tip the balance so as to shift the status of a cell from the healthy to the malignant phenotype thus giving rise to tumorigenesis (Figure 3).

Biological, chemical, and physical factors can all, either in combination or separately, lead to oxidative stress resulting in the production of reactive oxygen species (ROS) and/or reactive nitrogen species (RNS). Oxidative stress causes damage to tissues and elicits an immune response that induces inflammation in an attempt of the body to rectify the inflicted damage. During the inflammatory response, mast cells and leukocytes are recruited to the damaged site, leading to a “respiratory burst” because of an increased requirement and thus uptake of oxygen, hence increasing release and accumulation of ROS at the site of damage, a development further perpetuating the damage to the already damaged and inflamed site. As the level of inflammatory response is augmented, a series of signaling molecules are produced by the inflammatory cells such as metabolites of arachidonic acid, cytokines, and chemokines, acting to further intensify the inflammatory responses and the continuing production and accumulation of ROS.

Hence, oxidative stress increases in the microenvironment of the damaged site. Simultaneously, the signaling agents produced induce signaling cascades involving NF-κB, signal transducer and activator of transcription 3 (STAT3), HIF1-α, AP-1, nuclear factor of activated T-cells (NFAT) and Nrf2, which mediate immediate cellular stress responses. This includes induction of cyclooxygenase-2 (COX-2), inducible nitric oxide synthase (iNOS), aberrant expression of inflammatory cytokines: tumor necrosis factor α (TNF-α), interleukin-1 (IL-1), IL-6 and chemokines such as IL-8; CXC chemokine receptor 4 (CXCR4). This microenvironment now characterized by increasingly high levels of inflammation and oxidative stress can spread damage to neighboring cells/tissues and, if such condition is sustained and becomes chronic, it can eventually lead to carcinogenesis. Therefore, chronic inflammation, as mediated by the induction of the immune system due to oxidative stress, can predispose the host to cancer among various chronic illnesses [11,12,13,14,15,16].

Apart from the oxidative stress/inflammation axis and the related mechanism for induction of carcinogenesis, tumor cells activate autophagy in response to cellular stress and/or increased metabolic demands related to rapid cell proliferation [17]. Autophagy-related stress tolerance can induce cellular pro-survival mechanisms thus leading to tumor growth and therapeutic resistance. Interestingly, work with preclinical models, demonstrated that inhibition of autophagy can restore chemosensitivity and enhance tumor cell death thus improving response to therapy [17].

## 3. Risk Factors for Cancer

Although cancer is not exclusively a disease of the elderly, age constitutes one of the risk factors for cancer, as the majority of people diagnosed with the disease are 65 years or older. Certain habits/lifestyle choices are shown (epidemiologically) to increase risk of cancer, including use of tobacco and tobacco products (particularly smoking), drinking more than one alcoholic drink a day (for women of all ages and men older than age 65) or two drinks a day (for men age 65 and younger), excessive exposure to the sun or frequent blistering sunburns, and obesity [1,4]. A small, yet considerable proportion of cancers are due to an inherited condition. Although inherited genetic mutations do not necessarily lead to cancer development, cancer risk is increased, as the probability is significantly higher. The environment (natural and anthropogenic) may expose an individual to harmful substances, such as air pollutants, water pollutants, and various hazardous chemicals and other toxicants. Examples of chemical carcinogens are asbestos and benzene, and they are associated with an increased risk of cancer primarily of the lungs [1,4]. Exposure to carcinogens increases the risk of cancer at different degrees according to the type of carcinogen as the carcinogenicity/toxicity levels of compounds vary [18]. Infectious agents, such as viruses and bacteria increase cancer risk (e.g., human papillomavirus infections and higher risk of cervical cancer in women). Radiation, including ionizing radiation and non-ionizing radiation constitute risk factors for a variety of cancers [18]. Additional cancer risk factors include pharmaceutical agents and exogenous and endogenous hormones. Moreover, poor immune system status and inflammation, particularly chronic inflammation, are positively associated with cancer risk (e.g., *Helicobacter pylori* infection is strongly related to increased gastric cancer risk) [18].

Overweight/obese status is associated with an increased risk for many cancer types such as postmenopausal breast cancer, endometrial cancer, colorectal, esophageal, gallbladder, kidney, pancreatic and thyroid cancer [18]. Most of the molecules that are being investigated as potential mediators between obesity and cancer are cancer-promoting rather that cancer-causing per se, hence they do not interfere with DNA damage/correction and mutations; instead they induce growth and proliferation of malignant cells, while others are also involved in promoting metastasis [18]. However, the association between obesity and cancer is strong. According to data presented by National Cancer Institute (NCI) and the International Agency for Research on Cancer using European data, in 2002 alone 39% of endometrial cancers, 37% of esophageal cancers and 25% of kidney cancers were related to obesity. Moreover, the American Cancer Society, as reported in 2003, attributes 14% of all cancers in males and 20% of all cancers in females to excess weight [18]. Interestingly, on a population level, the overweight/obesity-attributable number of cancers is approximately equal to that attributable to current smoking. This surprising observation may be explained partly by the decreasing trends in smoking and simultaneously increasing trends for obesity. On an individual basis nevertheless, cancer risk due to smoking remains substantially higher than that attributed to obesity [18]. As per smoking, apart from first-hand smoking, second- and third-hand smoking also appear to contribute to increased risk for cancer, yet at a much lower rate [18]. 

A key environmental factor that interacts with the human organism at several levels is food/diet. Dietary constituents individually, synergistically and/or as a whole can influence the way the human body will respond from a biochemical perspective (i.e., metabolically), from a signaling perspective and from an epigenetic perspective among others. In this context, diet can be used as a tool to evoke the positive/desirable biological responses of an organism aiming to maximize health and protection against diseases (particularly chronic/non-communicable diseases) by mostly means of prevention.

Certain food groups such as fruits and vegetables among others have been significantly studied in this regard. Fruits and vegetables in particular have been suggested to exert cancer protective effects due to different mechanisms such as inhibition of carcinogen activation, stimulation of carcinogen detoxification, scavenging of free radical species, control of cell-cycle progression, inhibition of cell proliferation, induction of apoptosis, inhibition of the activity of oncogenes, inhibition of angiogenesis and metastasis, and inhibition of hormone or growth-factor activity [5,19].

## 4. Berries and Cancer

### 4.1. Rationale of the Current Review

Edible berries are being increasingly investigated for their potential to extend chemoprevention as well as protection against a variety of chronic diseases. Here we discuss the protective role of dietary berries in cancer. The focus of this review, regarding the selection of studies, was predominantly placed on in vivo and human studies. There is certainly an abundance of in vitro studies in the scientific literature where cell-lines and berry extracts are used to delineate the chemopreventive effects of berry extracts and/or specific compounds naturally found in berries at significant amounts. Even though these studies are indicative of the potential berries have in exerting favorable effects in cases of cancer, the systems are very specific and to a large extent notably simple and isolated from the physiological state. Therefore, these studies are often met with the criticism that they are limited in terms of their physiological significance and consequently exhibit limited applicability and translational potential. The reductionist approach and mechanistic studies along with logical reasoning have proposed some potentially key players in terms of chemoprevention and/or improvement of cancer prognosis in berries, however these compounds in isolation do not seem to extend the promised and desired effects optimally. It is highly uncertain which particular compounds extend the overall observed effects in humans. Most likely it is the synergistic action at varying degrees of contribution of a plethora of phytochemicals/bioactives complexly intervening at several molecular pathways. Therefore, from a nutritionist’s standpoint whole berry consumption is strongly advocated.

In this line of thought, our decision was to review and discuss the studies that were performed, either with intact animals or were done with humans, and where the form of the berries used was natural whole food. In vivo studies using freeze-dried berries are included as well. In terms of the berry types, our discussion was led by the availability of in vivo and human studies that used whole berries irrespective of what the berry itself was. We do present a limited number of characteristics from in vitro works that show bioactivity and chemoprevention potential for typical berry-found compounds as an indicator of potential mechanistic evidence for the mode of action towards chemoprevention and a lead-in to the core discussion.

A search for literature on in vivo and human studies investigating the effect of berry consumption on cancer initiation, progression, metastasis and overall risk was conducted. Relevant abstracts and full texts were screened. PubMed, Science Direct, Web of Science and Google Scholar databases were searched to identify articles published later than 1 January 2000. The searches used the following terms and text words alone and in combinations: “berry/berries”, “cancer”, “chemoprevention”, “cancer risk”, “anthocyanins”, “flavonoids”, “polyphenols”, “phytochemicals”, “animals”, and ‘humans’. Reference lists of the obtained articles were also searched for additional articles. The research was limited to English-language written articles. A total of 61 relevant articles were obtained from the database searches and from the reference lists of the obtained papers that met the aforementioned criteria. These studies were included in the review along with supportive data obtained from characteristic in vitro studies as well as evidence provided and discussed by recent review manuscripts. Additionally, we reviewed data from recent official reports issued by the World Health Organization, Center for Disease Control, and the European Commission. Here, we summarize the main results and conclusions, and discuss the findings of in vivo and human studies testing berries in terms of their anti-cancer properties.

### 4.2. Berry Types and Composition

According to strict botanical terminology a “berry” is a simple fruit with seeds and pulp produced from the ovary of a single flower and the pericarp (fruit wall) is fleshy (i.e., the fruit is fleshy from the skin layer inwards and all throughout to its core but the seeds) [20]. Under common usage however, the term berry refers to a small pulpy and often edible fruit. For example, blueberries and cranberries would be categorized as berries under both definitions, while bananas, tomatoes, grapes and pumpkin are berries according to the botanical definition, whereas blackberries, raspberries and strawberries, even though they are typically considered and referred to as berries by the lay audience, from a strictly botanical perspective they are not categorized as such. Hence, the term “berry” can indeed be fairly confusing since there are occasional discrepancies between the strict botanical criteria set, and what is widely used and/or accepted as being a berry. The studies included in this review refer mostly to fruits commonly consumed and recognized as berries.

**Figure 4:**
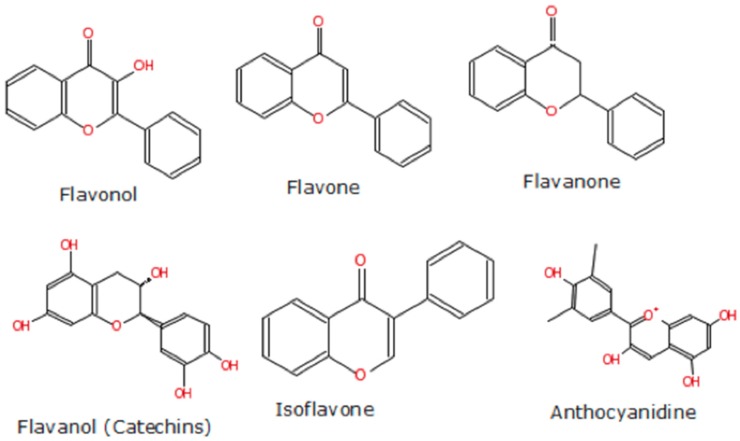
Major types of flavonoids (chemical structures produced via the eMolecules platform developed by eMolecules Inc., La Jolla, CA, USA).

**Figure 5:**
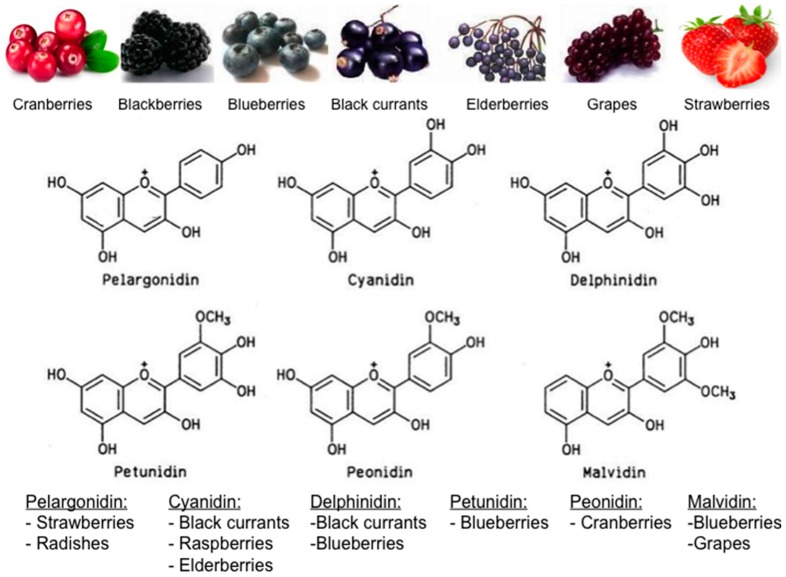
Major types of anthocyanins and characteristic examples of foods (berries) rich in the respective compounds (chemical structures produced via the eMolecules platform developed by eMolecules Inc., La Jolla, CA, USA).

Overall, berries are rich in polyphenols and most anthocyanins. Anthocyanins, along with other flavonoids, are localized in the skin, seeds and leaves of the berries giving them their distinctive pigmentation. Polyphenols constitute the largest group of phytochemicals found in plants, particularly in fruits, seeds, and leaves. There are more than 8000 chemical compounds found in the human diet today that are identified as dietary polyphenols. They are secondary metabolites of plants that contain one or more hydroxyl (–OH) group(s) attached to -ortho, -meta or -para position(s) on a typical benzene ring. These metabolites are generally involved in defense against ultraviolet radiation, various environmental pollutants, and pathogens [21]. Flavonoids on the other hand, constitute a very large (more than 6000 have been identified thus far) and very diverse group of phytonutrients (Figure 4 and Figure 5). Currently, even though a surplus of data from in vitro studies suggests antioxidant benefits from berries (particularly berry-extracts) due to several bioactive compounds—mostly polyphenols and flavonoids, (see Supplementary Materials Table S1)—a clear consensus stemming from in vivo and corroborated from human studies is missing. Health claims that foods containing polyphenols have antioxidant health value for consumers are not permitted on product labels by the official regulatory authorities in the USA (Food and Drug Administration, Silver Spring, MD, USA) or the European Union (EU-28), (European Food Safety Organization, Parma, Italy). Nonetheless, berries are widely considered functional foods that may well extend several health benefits.

### 4.3. Mechanisms Associated with Berries’ Anticancer Capacity

Edible berries are receiving increasing attention due to the great variety of phytochemicals, including but not limited to typical antioxidants, linked to protection against cancer among other chronic diseases. Berry constituents that have been suggested to exert cancer protective effects in cells include: phenolic acids (hydroxycynnamic acid, hydroxybenzoic acid), stilbenes (resveratrol, pterostilbene, piceatannol), flavonoids (anthocyanins, flavonols, catechins), lignans, tannins (proanthocyanidins, ellagitannins).

Berries are notably rich in compounds that are shown to exert potential for chemoprevention and particularly flavopiridol, ellagic acid, anethole and resveratrol have been demonstrated to inhibit the NF-κB signaling pathway, either at the point of signaling cascade activation or at the point of NF-κB’s translocation into the nucleus. Other points of interference include the DNA binding dimmers and/or interactions with the basal transcriptional machinery [22]. Resveratrol and anethole were also found to inhibit AP-1, which is linked to growth regulation and cell transformation. Further, AP-1 seems to be involved in the regulation of genes involved in apoptosis and proliferation while it can promote the transition of a tumor cell from epithelial to mesenchymal morphology, an early step marking metastasis [22]. Resveratrol and flavopiridol were demonstrated to down-regulate the expression of apoptosis suppressor proteins (Bcl-2 and Bcl-XL) in a variety of cancer cell-lines [22]. A variety of phytochemicals such as indole-3-carbinol, curcuminoids and epigallocatechin-3-gallate (EGCG) (as well as black raspberries as a whole fruit) have been shown to suppress Akt activation thus leading to cancer suppression signaling. Moreover, several common constituents of berries such as quercetin, kaempferol and pterostilbene were found to attenuate ROS in HepG2-C8 cells via the Nrf2-Antioxidant Response Element signaling pathway, suggesting that induction of antioxidant defense is likely one of the mechanisms via which berries provide chemoprevention [23]. Polyphenols are also likely to modify the redox state of cancer microenvironment thus rendering it more cytotoxic hence leading to apoptosis secondary to induced oxidative stress [24,25]. In various systems, berries have been shown to down-regulate all major inflammatory markers such as TNF-α, IL-1β, IL-6, IL-10, iNOS, COX-2, PGE_2_, NF-κB and p-p65 [24,25,26,27]. For example, anti-inflammatory pathways that have been shown induced by berry derived anthocyanin rich extracts include the reduction of expression levels of iNOS, COX-2, IL-1β and IL-6 in RAW264.7 macrophages [28]. In this regard, inflammation suppression may lead to protection against cancer occurrence and/or progression. The degree to which such protection is extended is yet unclear but appears significant. Additionally, berries are demonstrated to reduce cellular proliferation by down-regulating PCNA and Ki-67/MKI67, as well as inhibiting signaling pathways such as the PI3K/Akt/mTOR axis, MAPK/ERK and Wnt pathways [24]. Furthermore, anti-cancer effects can be extended via the inhibition of STAT3 and cell cycle arrest, thus impeding proliferation of cancer cells. Hence, cancer inhibition of cellular growth and proliferation may function towards deceleration of cancer progression thus improving the possibility for anti-cancer treatment success and subsequent patient’s survival. Another potential mechanism via which berries were shown to confer protection against cancer development is the induction of apoptosis. Berries may confer chemoprevention via the activation of caspases and the mitochondrial damage/cytochrome c pathways hence leading to enhanced apoptosis of cancer cells [23]. Berries were observed to up-regulate p53, caspases-3, -8 and -9 as well as inducing Bax and Cyto-c while down-regulating Bcl-2 and PARP [24,25]. Therefore the progression of cancer can be decelerated thus assisting the treatment efforts, consequently improving survival rates of patients. Cell cycle regulation also appears to constitute yet another potential mechanism through which berries may exert their anticancer function. More specifically, berries were found to increase p16, p21 and p27 while reduce a series of cell cycle markers such as CDK_2_, CDK_4_, cyclin A, cyclin B1 and cyclin D1, Cdc2, Cdc25C and the Rb protein [24]. Such synergies make a cell less prone to cancerous transformation and reduce the potential for tumorigenesis. The reduction of angiogenesis has also been proposed as a potential mechanism of berries’ function. A series of pro-angiogenic factors such as c-Myc, c-jun, c-fos and VEGF, have been shown markedly reduced by berries [24]. Cell adhesion proteins such as β-catenin, ICAM-1 and VCAM-1 are attenuated by berries [24], while cellular invasion is also inhibited through the repression of MMP and u-PA by anthocyanins commonly found in berries [29]. These observations underline cell adhesion attenuation and cellular invasion suppression as additional potential mechanisms via which berries may provide anti-cancer protection. As for cell adhesion, invasion and migration along with angiogenesis are all phenomena that facilitate metastasis, berries may extend inhibition of metastasis by suppressing such pro-metastatic phenomena. Additionally, effects on phase-I and -II enzymes have been proposed as potential mechanisms of berries’ anti-cancer function [29]. More specifically, polyphenols have been shown to inhibit strongly phase-I detoxifying enzyme CYP1A1 while inducing phase-II detoxifying enzymes GST and NADPH quinone oxidoreductase (NQO) in mouse epidermal cells [29].

Furthermore, other modes of berry activity via certain bioactive compounds they contain involve modulation of miRNA expression profiles, DNA methylation and histone modifications, all of which can lead to the inhibition of cancer cell growth, induction of apoptosis, reversal of epithelial-mesenchymal transition, or improvement of conventional cancer therapeutics’ efficacy [30]. Epigenetic alterations such as inhibition of histone deacetylases (HDACs), miRNAs and modification of the CpG methylation of cancer-related genes may indeed be key mechanisms by which berries reduce cancer risk, especially when taking into account the significantly lower concentrations of bioactive compounds in human blood compared to in vitro testing [23,30]. A plausible explanation for their activity in humans, even at markedly lower concentrations, could be that these compounds exert their biological activities through epigenetic modulation. There is growing interest in dietary compounds that confer favorable epigenetic modulation against chronic diseases such as cancer. Characteristic is the example of resveratrol, which activates class III HDACs (sirtuins) because of their potential role in extending lifespan and in reducing, or delaying, age-related diseases including cancers [30]. A summary of the mechanisms through which berries may extend chemoprevention and/or evoke therapeutic responses post-cancer occurrence is provided in Table 2. Below we discuss evidence from studies indicating anti-cancer function of specific bioactive compounds typically found in berries.

### 4.4. Berries’ Potential for Cancer Risk Reduction Due to Specific Constituents

A plethora of berry-derived compounds has been studied for their chemopreventive properties [31], but also as a proxy for establishing potential mechanisms via which berries may be extending chemoprevention as suggested by epidemiology. The major phenolics isolated from the apple and berry juice, such as phlorizin, rutin, quercetin and their two glucoside forms, and phloretin, exhibited significant inhibition of cytochrome’s P4501A1 enzymatic activity [32]. Kern and colleagues showed that the activity of protein kinase C (PKC), a well-established signaling molecule involved in carcinogenesis induction, is significantly reduced in HT29 cells when incubated with polyphenol-rich apple juice extract (AE02) for 24 h although the result was not sustained. More interestingly, prolonged incubation of HT29 cells with AE02 resulted in significantly induced apoptosis via the activation of caspase-3, DNA fragmentation, and cleavage of poly(ADP ribose) polymerase [26,33]. Wang and Jiao [26] showed that ROS such as super-oxide radical and hydrogen peroxide (H_2_O_2_), were found to be sequestered by different berry-juices (i.e., blackberry, strawberry, raspberry, cranberry and blueberry), thus offering a potential mechanism for cancer risk reduction. Besides the direct scavenging ability of berries, their antioxidant potential due to the polyphenols they contain is also an element that further enhances their anti-cancer properties. Additionally, antioxidant enzymes such as catalase, glutathione reductase and ascorbate peroxidase were identified in significant concentrations in strawberries and blackberries thus offering yet another potential means of cancer chemoprevention extended by berries. The combination of anti-ROS elements in berries could mount a resistance at the very first level/step of carcinogenesis; ROS-induced DNA damage. On the other hand, DNA repair seems to be promoted through the induction of oxidative adducts’ removal from the DNA at least in some cases. In a study with mice, administration of ferulic acid, a phenolic acid commonly found in berries, was demonstrated to significantly reduce DNA strand breaks after whole body γ-irradiation [34]. Berries (i.e., strawberries, black raspberries and blackberries) in their lyophilized form as part of diet in rats were shown to significantly inhibit the appearance and development of carcinogen-induced tumors [35].

In a review of the evidence comprised by Lall and colleagues, the relationship between polyphenols and prostate cancer was examined. Although the results are occasionally inconsistent and somewhat variable, there is a consensus that polyphenols constitute promising agents for the management of prostate cancer. Gallic acid (GA) for example has been shown to exert anti-cancer function in human PCa DU145 [21] and B16 melanoma [36] cells. In separate studies with nude mice, GA effectively inhibited growth of tumor in both DU145 and 22Rν1 prostate cancer xenografts, while it successfully decreased micro-vessel density, as compared to controls [37].

A series of in vitro experiments with HCT-15 intestinal carcinoma cells, demonstrated that growth was effectively inhibited by anthocyanin fractions extracted from different cherry and berry extracts in comparison to that of flavonoid fractions [38] while berry extracts including lingonberry, strawberry, blueberry, and bilberry extracts that contain anthocyanins inhibited the growth of HCT-116 colon cancer cells. Studies with Caco-2 colon cancer and HT-29 colonic crypt resembling cells demonstrated that flavonoids can significantly reduce proliferation. Dosing studies for the flavonoids showed anti-proliferative activity of all compounds with EC50 values ranging between 39.7 ± 2.3 microM (baicalein) and 203.6 ± 15.5 microM (diosmin) [39]. The anti-oxidant activity of the raspberry was directly related to the total amount of phenolics and flavonoids found in the raspberry (*p* < 0.01). No relationship was found between anti-proliferative activity and the total amount of phenolics/flavonoids found in the same raspberry (*p* > 0.05) suggesting that anti-proliferative capacity is maxed-out at a certain level of phenolics/flavonoids exerting their function, possibly through molecular signaling [40].

In terms of the form of berries, it has been demonstrated that wild grown species generally contain more phenolics than cultivated ones [41]. Measurement of the antioxidant activity of anthocyanin extracts in the case of blueberries for example, showed that there was no significant difference between fresh, dried, and frozen blueberries suggesting that the major compounds of interest are relatively stable and unaffected by general food industry typical processes and relevant production methods [42].

## 5. The Role of Dietary Berries in Various Types of Cancer

### 5.1. Cancers of the GI Tract

#### 5.1.1. Diet and Colon Cancer—Berries/Phenolics and Colon Cancer

A close link is observed between colon carcinogenesis and chronic inflammation of the intestine in mouse models of inflammation and cancer [43,44,45,46].

Encouraging results from human studies pave the road for further clinical studies with black raspberry/berries especially considering the possibility of formulating berry-derived foods with consistent bioactive composition that can also be scaled-up for large clinical trials [47]. Berry phenolics, despite extensive metabolism and structural changes, seem to maintain their protective effects in relation to colon carcinogenesis [48].

##### In Vivo Studies

Protective effects of the mulberry fruit, containing flavonoids and anthocyanins, alkaloids and carotenoids, were documented by in vitro and in vivo studies on colon cancer and intestinal inflammation [49]. In MUC2(−/−) mice, a model of spontaneous chronic intestinal inflammation at an early age and intestinal tumors at three months, a diet enriched with 5% or 10% mulberry extract administered for three months, starting at 3–4 weeks of age, elicited a reduction in tumorigenesis and intestinal inflammation as estimated by the degree of mucosal damage and lymphocyte infiltration [49]. In the same study, 6–8 week old BALB/c mice supplemented with mulberry extract (5% or 10% wt/wt) for 10 days before exposure to 3% dextran sulfate sodium (DSS) in drinking water for 9 days, showed an improvement in typical symptoms of DSS-induced acute colitis such as weight loss, bloody stools and colorectal histological changes [49]. In lipopolysaccharide (LPS)-treated RAW264.7 macrophages, mulberry extracts attenuated inflammation by reducing the expression of iNOS, COX-2, IL-1 beta and IL-6, and inhibiting activation of NF-κB/p65 and pERK/MAPK pathways [49]. Strawberry-enriched diets for 13 weeks were documented to inhibit chemically induced colorectal cancer in Crj: CD-1 mice after treatment with azoxymethane (AOM) and dextran sulfate sodium (DSS). Tumor incidence of the mice fed with freeze-dried strawberry powder at 2.5%, 5% and 10% (wt/wt) of the diet was measured at 64%, 75% and 44%, respectively, versus the 100% of their littermates fed the control diet. Tumor multiplicity was also reduced in all strawberry-fed mice, but reached statistically significant difference only with the 10% strawberry diet. Colon inflammation was also reduced due to the strawberry diet as observed by decreased nitrotyrosine, phosphorylation of PI3-kinase, Akt, ERK and NF-κB, expression of TNF-α, IL-1β, IL-6, iNOS and COX-2, as well as activity of iNOS and COX-2 [46]. 

Both animal and human studies have documented the anti-carcinogenic properties of bilberry anthocyanins [50,51]. In ApcMin mice, model of human familial adenomatous polyposis, a condition characterized by multiple intestinal polyps potentially developing to carcinomas, an anthocyanin-rich bilberry extract (40% anthocyanins) fed for 12 weeks at a dietary dose of 0.3% elicited a 30% reduction o adenoma counts in a dose-dependent manner [50].

In a pilot study, a standardized bilberry extract (36% anthocyanins wt/wt) was administered daily to 25 colorectal cancer patients for 7 days before scheduled resection of primary tumor or liver metastases. All the three different doses provided—1.4, 2.8, or 5.6 g (0.5–2.0 g anthocyanins)—were safe and well tolerated. Compared to pre-intervention, a significant 7% decrease of the proliferation index was observed in all colorectal tumors from all patients. In the lowest dose to patients, a significant 9% decrease in tumor tissue proliferation was measured, while with the other doses the observed decrease was not significant. In addition, serum insulin-like growth factor-1 levels were lower than pre-intervention in all patients, although not significantly [51]. Similarly, animal dietary studies with black raspberries reveal the chemopreventive capacities of the fruit in models of ulcerative colitis, several rodent models of colon cancer and adenoma, as well as humans [52,53,54,55,56,57,58].

Interleukin-10 knockout mice, model of ulcerative colitis-associated colon cancer progression, when fed with 5% wt/wt black raspberry presented diminished colon ulceration, along with lower levels of β-catenin nuclear translocation and inhibition of epigenetic events related to abnormal Wnt signaling [53]. Similarly, in DSS-induced ulcerative colitis in C57BL/6J mice, a 5% wt/wt black raspberry diet suppressed colon ulceration by restoring epigenetic regulation of systemic inflammation [59]. Intestinal tumor formation and cell proliferation was inhibited after a 12-week consumption of a Western-style diet supplemented with 10% (wt/wt) freeze-dried black raspberries in Apc1638+/− and Muc2−/− mice, both models of human colorectal cancer, although via distinct mechanisms. Tumor incidence and multiplicity was reduced by 45% and 60%, respectively in the Apc1638+/− mouse, and by 50% in the Muc2−/− mouse. A slight, non-significant reduction of tumor size was also observed. Signaling mediated by β-catenin and chronic inflammation, leading to colon pathology in the Apc and Muc2−/− mouse, respectively, were both attenuated due to the black raspberry diet. In the Apc1638+/− mouse, β-catenin was markedly reduced, while its downstream effectors, c-Myc and cyclin D1 were slightly reduced, along a positive, but non-significant modulation of several inflammatory markers (COX-2, TNF-α, IL-6, IL-10 and IL-1) [52].

A 5% wt/wt black raspberry supplementation for 8 weeks reduced polyp number and size in the Apc(Min/+) mice intestine and colon, inhibiting the development of colonic adenoma. Non-targeted metabolomics revealed that several apc-related metabolites in the mucosa, liver and feces were positively modulated by the black raspberry diet. Among the affected metabolites, putrescine and linolenate, are associated with colorectal cancer in humans [53]. In Fischer 344 rats consuming black raspberries at 2.5%, 5%, or 10% (wt/wt) of diet after AOM injections, aberrant crypt foci (ACF), total tumor and adenocarcinoma multiplicity were significantly reduced at varying percentages depending on the berry dose, while tumor burden showed a non-significant decrease across all berry diet groups. With regards to the dietary dose of black raspberries, the 5% dose was more effective than the 2.5% dose in inhibiting chemically induced adenocarcinoma, while the 10% dose did not produce any greater benefit. Finally, oxidative stress, as measured by urinary levels of 8-hydroxy-2′-deoxyguanosine (8-OHdG), was attenuated significantly in all rats supplemented with black raspberries [54].

The chemoprevention effect of three types of berries (bilberry, lingonberry and cloudberry) on intestinal tumorigenesis was tested in Min-mice by Misikangas et al. A diet containing 10% (wt/wt) freeze-dried bilberry, lingonberry or cloudberry fed for 10 weeks to mice produced a 10%–30% (statistically significant) reduction of intestinal carcinomas as well as reduced tumor burden by more than 60% [59]. As assessed by microarray analyses, with berry enriched diets the authors observed attenuation of genes implicated in colon carcinogenesis, including the decreased expression of the adenosine deaminase, ecto-5′-nucleotidase, and prostaglandin E2 receptor subtype EP4, thus suggesting some mechanistic evidence as to a potential explanation for the tumor risk reduction. In separate in vivo experiments also on Min mice, Rajakangas et al., showed that a 10% white currant dietary supplementation administered for 10 weeks can yield a significant reduction in the number and size of adenomas in the total small intestine, associated with reduced nuclear beta-catenin and NF-κB protein levels in the adenomas [60].

Many flavonoids have been shown to exert anti-carcinogenic effects in cells and animals. In many of these cases the compounds are administered in isolation or as part of a whole food yet processed, usually freeze-dried. Many of the chemopreventive effects observed seem to occur through signaling pathways known to be important in the pathogenesis of colorectal, gastric and esophageal cancers. Nonetheless, dietary flavonoid intakes are generally low and their metabolism in humans is extremely complex [61]. Additionally, the amounts and the optimum mixture of these protective compounds are unclear. Certain antioxidants exert undesirable pro-oxidant action when the dosing is high. As far as the initiation and progression of cancers of the gastrointestinal (GI) tract in humans, it is more probable that any adverse effects of diet are caused primarily by over-consumption of energy, coupled with inadequate intakes of protective substances, including micronutrients, dietary fiber and various phytochemicals. Carcinomas of the esophagus, stomach and colon all are suggested based on epidemiological observations to be partially preventable by diets rich in fruits and vegetables [62,63]. Hence, the consumption of berries is advisable in the context of risk reduction for colon cancer.

##### Human Studies

There is a limited number of human studies investigating the effect of edible berries on colon cancer, nevertheless the available clinical results are encouraging overall, indicating potential health benefits not only in terms of chemoprevention but also in regards to cancer patients’ responses. More specifically, black raspberry was documented to reduce significantly cancer cell proliferation in 20 cancer patients, 6 with colon cancer and 14 with rectal cancer; 17 male and 3 female [56]. The study participants consumed three times daily 20 g of freeze-dried berry powder mixed with 100 mL water for varying periods of 1 to 9 weeks (4 weeks on average) [56]. It is estimated that daily consumption of 60 g powder is equivalent to 0.59 kg of fresh black raspberry and a rodent diet of approximately 7% wt/wt powder, a dose adequate for chemoprevention in animal studies [63]. The black raspberry treatment modified positively genetic and epigenetic markers measured in colorectal adenocarcinomas and adjacent normal tissues, as observed by modified gene expression of β-catenin and E-cadherin downstream of the Wnt pathway, and demethylation of *SFRP2* and *WIF*, tumor suppressor genes upstream of the Wnt pathway [56]. Aberrant signaling of Wnt/β-catenin pathway occurs in about 85% of sporadic colorectal cancers and is mainly attributed to Apc gene mutations [64]. In addition to Wnt pathway, black raspberry modified protectively expression of genes related to proliferation, apoptosis, and angiogenesis [56]. In 24 cancer patients receiving the same black raspberry treatment, plasma concentration of granulocyte macrophage colony stimulating factor (GM-CSF) was increased compared to measurements before initiating the berry treatment, while the other 8 plasma cytokines measured (IL-1β, IL-2, IL-6, IL-8, IL-10, IL-12p70, Interferon-γ, TNF-α) were not affected significantly. However, plasma concentrations of IL-8 decreased when the berry drink was consumed for longer than 10 days, suggesting that longer interventions may be needed for greater changes to occur [57]. In the same cohort of cancer patients non-targeted metabolomics in urine and plasma samples, showed significant changes in a number of metabolites (34 and 6, respectively) related to energy pathways, including increased carbohydrate and amino acid metabolites [57]. The black raspberry extract, which was reasonably well tolerated by cancer patients, overall showed promise as an ameliorating strategy in colon cancer cases.

Animal and human studies of two commercial products containing blackcurrant extract power, with or without lactoferrin and lutein, demonstrated protective effects of the berry supplement on the colonic microbiota, including changes in bacterial population and pH associated with colon cancer risk [65,66]. Sprague-Dawley rats, starting at 8 weeks of age, were fed the blackcurrant extract (13.4 mg/kg of body weight) three times weekly for a period of 4 weeks. A significant increase in beneficial populations of lactobacilli and bifidobacteria, along with increased activity of the related enzyme β-glucosidase, was attributed to the berry extract. In addition, the berry extract was shown to reduce bacteroides and clostridia, as well as the activity of β-glucuronidase, a bacterial enzyme involved in colon carcinogenesis [65]. Similarly, in thirty healthy volunteers (16 women, 14 men, age 20–60 years) the blackcurrant extract (672 mg/d for two weeks) elicited a positive modulation of gut microbiota by enhancing growth of lactobacilli and bifidobacteria (beneficial microflora), while lowering fecal pH, bacteroides and clostridia and inhibiting β-glucuronidase [66].

### 5.2. Esophageal Cancer

A significant amount of data, derived from in vivo work, demonstrate a potential set of benefits from berry consumption in terms of both chemoprevention and response to cancer in the case of esophageal cancer. Stoner and colleagues described the effects of black raspberries on gene expression in the very early stages of rat esophageal carcinogenesis and particularly showed effects on genes that influence carcinogen metabolism. More specifically, out of 2261 dysregulated genes in the esophagi of Fisher-344 rats treated for one week with nitrosomethylbenzylamine (NMBA), 462 were positively modulated and restored back to near-normal levels when a 5% (wt/wt) whole black raspberry (BRB) powder supplementation to the control AIN-76A diet was introduced [67]. The genes that were positively regulated were oncogenes, genes involved in oxidative damage and tumor suppressor genes regulating apoptosis, cell cycling and angiogenesis [67]. Furthermore, in other feeding experiments with F344 rats, animals were fed berry-supplemented diets for 2 weeks prior to NMBA treatments, and were then maintained on the berry-supplemented diets until the end of the 30-week experiment at which point esophageal tumors were counted. The rats were fed 5% (wt/wt) BRB powder, an anthocyanin-rich fraction, an organic solvent-soluble extract (each contained approximately 3.8 μmol anthocyanins/g diet), an organic-insoluble (residue) fraction (containing 0.02 μmol anthocyanins/g diet), a hexane extract, and a sugar fraction. The experiments indicated that anthocyanins derived from black raspberries were effective in reducing NMBA-induced tumors in the rat esophagus [68]. While several forms of anthocyanins were evaluated, all forms, i.e., 5% whole raspberries extract, anthocyanin-rich fraction and organic-solvent soluble extract, produced similar results. Interestingly, the organic insoluble residue fraction also produced comparable results, hence indicating that other compounds from berry anthocyanins may also extend a chemopreventive effect [68]. 

Alteration in the immune cell trafficking in esophageal cancer by anthocyanin constituents was shown in a rat feeding study [69]. After 5 weeks of NMBA (0.35 mg/kg) administration while rats were all on control diet, the animals were switched to the treatment diets (i.e., 6.1% black raspberries powder, anthocyanin-rich fraction of BRB 3.8 μmol/g, and 500 ppm protocatechuic acid). Esophageal cancer-related inflammatory biomarkers were assessed at three time-points (15, 25 and 35 weeks) in the plasma and at the esophagus. Furthermore, the infiltration of the immune cells in the esophagus was also evaluated. The production of cytokines was not different among all three different dietary treatments. However, all treatment groups exhibited a decrease of IL-1β and IL-12 and an increase of IL-10 compared to the control, while the treatments all produced a significantly lower infiltration of both macrophages and neutrophils to the esophagus. These data, taken together, suggest that anthocyanins inhibit esophageal tumorigenesis by altering cytokine expression and innate immune cell trafficking in the tumors [69].

The effect of black raspberries (whole food) in the late stages of rat esophageal carcinogenesis was investigated thoroughly by Wang and colleagues [70]. A 5% (wt/wt) freeze-dried raspberry-supplemented diet was evaluated on F344 rats versus control diet in terms of its influence in the late stages of carcinogenesis for NMBA-induced esophageal cancer. Rats were evaluated 35 weeks post-NMBA treatment and while on control versus raspberry-supplemented diets. Results showed that BRB reduced the number of dysplastic lesions as well as both the number and size of esophageal tumors. Furthermore, BRB was found to have a positive impact on the expression of a variety of genes associated with pre-neoplastic esophagus as well as esophageal papillomas, and found to modulate gene expression associated with proliferation, apoptosis and inflammation as well as angiogenesis in a positive manner. These results demonstrate that BRB can have a positive impact in terms of molecular signaling in the cases of even late stages carcinogenesis in esophageal cancer in rats, thus underlining potential benefits from black raspberry consumption in terms of cancer prevention and/or treatment.

Further evidence on the positive role of black raspberry consumption was provided by similar experiments conducted by Chen and colleagues [71]. A 5% (wt/wt) BRB diet was shown to reduce both mRNA and protein levels of COX-2, iNOS and c-Jun as well as level of prostaglandin E2 in F344 NMBA-treated rats on diets for 25 weeks. These findings suggest another potential role in cancer molecular signaling and possible therapeutic strategy in the case of esophageal cancer for black raspberries. The same group also reports a BRB angiogenesis-suppression parallel to the suppression of COX-2 and iNOS in separate experiments [72]. Other researchers observed similar positive effects of BRB in NMBA-induced esophageal cancer in F344 rats although the effects were not dose-dependent (5% and 10% wt/wt BRB supplementation produced similar effects) [73]. Such observations support the notion that finest dosing is optimizing health benefit, probably primarily through molecular signaling and hence more emphasis needs to be placed on the determination of the “ideal” mixture of bioactive compounds and dosing when it comes to dietary advice. 

Stoner et al., [74] assessed the chemopreventive ability against esophageal cancer in rats, for a variety of berries. NMBA-treated rats over a period of 5 weeks were placed on diets supplemented with 5% (wt/wt) freeze-dried black or red raspberries, strawberries, blueberries, noni, açaí or wolfberry. The diets were provided for 35 weeks. The study revealed that all berry types were about equally effective in inhibiting NMBA-induced tumorigenesis in the rat esophagus although the content of anthocyanins and other comparable phytochemicals varies significantly among these berries. Interestingly, in all cases, the levels of serum cytokines, interleukin 5 (IL-5) and GRO/KC, the rat homologue for human interleukin-8 (IL-8) were notably reduced. These observations suggest that potential berry chemoprevention may be exerted via a variety of combinations in terms of type and amount of bioactive compounds thus not only underlining the importance of quality and synergism but also the importance of overall diet (as a pattern) in terms of diet-associated health benefits.

Kresty and colleagues demonstrated that cranberry proanthocyanidins inhibit esophageal adenocarcinoma both in vitro and in vivo. Specifically, purified cranberry-derived proanthocyanidin extract (C-PAC) was evaluated in its capacity to extend chemoprevention utilizing acid-sensitive and acid-resistant human esophageal adenocarcinoma (EAC) cell lines and esophageal tumor xenografts in athymic NU/NU mice [75]. Results showed pleiotropic cell death induction and PI3K/AKT/mTOR axis inactivation upon in vitro and in vivo exposure to C-PAC, hence indicating a potential mode of action for C-PAC chemoprevention.

### 5.3. Breast Cancer

Berry potential in reducing risk of breast cancer has been demonstrated by in vivo studies. More specifically, the preventive and therapeutic potential of highbush blueberry was investigated in (August-Copenhagen-Irish) ACI female rats supplemented with 5% wt/wt blueberry, 2 weeks before or 12 weeks after treatment with the carcinogen 17β-estradiol (E2), respectively. The tumor latency for palpable mammary tumors was delayed, while tumor volume and multiplicity was reduced in both intervention modes. A smaller dose of 2.5% blueberry diet administered after E2 treatment (i.e., therapeutically) in another experimental group also attenuated tumor multiplicity [76,77]. The aforementioned anti-tumor effects of blueberries were in agreement with favorable molecular changes such as down-regulation of CYP1A1 and ER-α gene expression, controlling E2 metabolism and signaling, respectively [76,77]. Blueberry and black raspberry were shown to possess protective effects against estrogen-induced breast cancer, although with varying impact and potential mechanism. At 5% wt/wt of diet, blueberry appeared more effective in reducing tissue proliferation, tumor burden and down-regulating CYP1A1 expression, while black raspberry delayed tumor latency to a greater extent and down-regulated ERα expression [77].

Notably, a series of phytochemicals abundantly found in berries such as cyanidin, delphinidin, quercetin, kaempferol, ellagic acid, resveratrol, and pterostilbene have been shown by in vitro and in vivo studies to interact and interfere with key pathways in breast cancer as well as induce apoptosis and autophagy thus reducing risk for breast cancer development and recurrence [78].

### 5.4. Miscellaneous Cancers and Berries

In addition to the most studied cancers (those of the GI tract and hormonal cancers), in terms of the role of berries in risk attenuation, impressive evidence is accumulating, suggesting that consumption of berries may well offer chemoprevention and/or improve responses in several types/cases of cancer. Several studies have demonstrated that strawberry consumption in rodents exerts chemoprevention in a series of cancers including oral cavity, breast, lung and esophageal [79,80,81,82,83]. In a phase II clinical trial strawberry consumption (60 g/day for 6 months) inhibited the progression of precancerous lesions [84]. Based on previous observations, potential mechanisms of anticancer activity include suppression of NF-κB, COX-2 and iNOS. Some evidence support a potential positive role of berries (specifically dietary polyphenols) in the case of prostate cancer [21] but there is no definitive conclusion as to the mechanisms of action, while results are sometimes inconsistent and variable.

Using the hamster cheek pouch model, Casto and colleagues investigated the potential of lyophilized strawberries to inhibit tumorigenesis. More specifically, animals were painted three times a week for six weeks with 0.2% 7,12-dimethylbenz(a)anthracene (DMBA) to induce oral cancer [79]. Hamsters were given diets supplemented with 5% or 10% wt/wt lyophilized strawberries prior to, during, and after, or only after carcinogen treatment. At 12 weeks after DMBA initiation, animals were terminated and number of lesions and tumors was counted [79]. Results revealed significantly fewer tumors and lesions in the strawberry-supplemented diet groups compared to controls, suggesting a potential positive effect of strawberries in the case of oral cancer. In separate experiments using the same animal model and carcinogen Zhu et al., demonstrated that tumor incidence, multiplicity, volume and histological grade of oral precancerous lesions were reduced in hamsters fed a 5% wt/wt lyophilized strawberry supplemented diet compared to animals fed control diet [84]. From a mechanistic perspective, the study showed that strawberries suppress cell proliferation, angiogenesis and oncogenic signaling and arachidonic acid metabolism [84].

Research conducted by Bishayee et al., showed that in rats fed a diet supplemented with pomegranate extract, PE (1 or 10 g/kg) 4 weeks before and 18 weeks following diethylnitrosamine (DENA)-initiated hepatocarcinogenesis, PE dose-dependently suppressed a series of elevated inflammatory markers (COX-2, NF-κB) [85].

Evidence to suggest that berry extracts can yield chemoprevention against lung cancer was provided by Balansky et al. Whole-body Swiss ICR mice were exposed to mainstream cigarette smoke, at birth and then daily for 4 months. Black chokeberry and strawberry aqueous extracts were given as the only source of drinking water, starting after weaning and continuing for 7 months. Both berry extracts inhibited lung adenomas’ development [81].

Knobloch and co-workers in a phase 0 human study showed that black raspberries, when administered as freeze-dried powder supplement in oral torches to oral cancer biopsy-confirmed patients, improve gene expression profile associated with oral cancer [86]. Transcriptional biomarkers assessed showed significant improvement for the berry supplemented diet demonstrating marked reduction for the pro-inflammatory genes NFKB1, PTGS2 as well as pro-survival genes AURKA, BIRC5, EGFR all strongly associated with oral cancer risk and progression [86].

Work performed by Mallery et al., showed that in patients with oral intraepithelial neoplasia lesions, topical application of bioadhesive gels that contained 10% wt/wt freeze-dried black raspberries for 12 weeks resulted in statistically significant reduction in lesional sizes, histological grades, and loss of heterozygosity events compared to placebo controls [87].

In a data analysis of a case-control human study of 230 patients conducted in Italy, a favorable role of flavonoids and proanthocyanidins in gastric cancer was demonstrated by Rossi and colleagues [88]. In separate studies, Rossi et al., conducted a cohort study in Northern Italy between 1991 and 2008 with 326 cases of incident pancreatic cancer and respective controls, examining the relationship between flavonoids and pancreatic cancer risk. According to their results the analyses showed that dietary proanthocyanidins (mostly present in apples, pears and pulses), may convey some protection against pancreatic cancer risk [89].

The potential effect of diets on cancer risk has been studied widely. Even though there is no specific and defined diet that reduces cancer risk, diets rich in fruits and vegetables have been repeatedly found to be beneficial in terms of reducing cancer risk. In this context, the scientific consensus refers more to dietary habits or good dietary practices than a well-defined diet per se. However, there has been notable amount of evidence to suggest that adherence to a Mediterranean diet is associated with reduced risk of overall cancer mortality as well as reduced risk of incidence of several cancer types (especially cancers of the colorectum, GI tract, breast, stomach, pancreas, prostate, liver) according to observational studies [90,91]. Interestingly, the Mediterranean diet is a diet rich in fiber, fruits, vegetables and grains as well as olive oil and fish while from a compound standpoint rich in vitamins, antioxidants, flavonoids, anthocyanins, and other phytochemicals as well as ω-3 fatty acids. Furthermore, notably the Mediterranean diet is often viewed as a set of practices and a life-style much more so than a mere diet or dietary scheme. Interestingly, a study showed that if fruit and vegetable intake levels increased to 300 and 400 g/day respectively, that could lead to a significant reduction in gastric cancers, particularly in the developing countries [92].

Even though the mechanisms remain not completely clarified, there is convincing evidence from epidemiological and experimental studies that dietary factors are likely to have a major influence on the risk of several types of cancer, particularly of the GI tract [93,94,95,96,97]. In vivo experiments using mice showed that diet-induced obesity increases risk of colonic cancer while a signaling/modular implication of leptin (both a hormone and a cytokine) was also suggested by the data [96,97]. In a more global approach, berries have been shown to play a positive role in health protection in cases of chronic diseases other than cancer as well, where inflammation and oxidative stress are key initiators for the onset of a disease [98,99,100]. In this regard, placing more emphasis on the dietary constituents but also on the whole food and on dietary patterns that may confer health benefits and potential protection against chronic diseases is important.

## 6. Conclusions

Although the initiation, progression and development of cancer is a multi-factorial phenomenon, the contribution of diet to chemoprevention has been suggested by a bulk of evidence stemming from in vitro, in vivo and human studies. A particular interest has been placed on fruits and vegetables of which edible berries constitute an interesting sub-group, as they are rich in a variety of compounds that have been shown to exert favorable effects in several types of cancers. Anthocyanins, flavonoids, other antioxidants and a plethora of more or less well-defined phytochemicals with antioxidant and other properties seem to offer a notable arsenal that reduces risk of cancer as evidenced by in vivo and human work. The mechanistic details of the mode of action remain somewhat elusive although involvement of certain pathways has been demonstrated. These pathways are responsible for the modulation of different cellular processes, showing certain common signaling events such as arrest of cell cycle by increasing levels of cyclin-dependent kinase inhibitor proteins (CDIs) and inhibition of cyclins, induction of apoptosis via cytochrome c release, activation of caspases and down- or up-regulation of Bcl-2 family members, inhibition of survival/proliferation signals (Akt, MAPK, NF-κB) and inflammation (COX-2, TNF, IL secretion), as well as suppression of key proteins that are critically involved in angiogenesis and metastasis.

A significant amount of work to identify and delineate mechanistic details has been undertaken in cell lines, which although useful and indicative, must be corroborated by in vivo data. Thus in vitro data interpretation must be careful and cautiously extrapolated to the in vivo and much more so to the human level. The work we have summarized herein, primarily from in vivo and human studies, indicates favorable effects of berry consumption in a variety of cancers by mechanisms that involve oxidative stress, inflammation and the related signaling. It appears rather doubtful that a particular compound in berries is responsible for the health benefits these food items extend. It seems that whole food consumption (edible berries) may be optimizing the benefit arguably due to synergies. Furthermore, it is highly challenging to decipher and mimic the exact mixture of berry compounds, quality/types of compounds and amounts, or physicochemical conditions and matrix involvement in berries that confer the benefit.

Edible berries have been demonstrated to extend chemoprevention in cancer primarily of the GI tract as well as breast and to a lesser degree of liver, prostate, pancreas and lung. Notably, no negative effects have been reported by berry administration, thus making it a plausible and potentially useful dietary strategy to reduce risk of cancer and help cancer patients with disease prognosis.

## Figures and Tables

**Table 1:** Deaths by major types of cancer in 2012 (world).

Type of Cancer	Deaths (Figures in Millions)
Lung	1.590
Liver	0.745
Stomach	0.723
Colorectal	0.694
Breast	0.521
Esophageal	0.400
Total	4.673

Source of data: WHO—world cancer report 2014.

**Table 2:** Summary table of mechanisms and mode of action via which berries may evoke chemopreventive and therapeutic responses against cancer.

Mechanism	Mode of Action	Bioactive Compound *
Anti-oxidative action	ROS sequestration ↑ GSH	Epigallocatechin-3-gallate (EGCG)
Effects on enzymes of phase-I and -II	↓ CYP1A1 ↓ LDH	Quercetin, kaempferol
	↑ UDPGT ↑ NQO	Ellagic acid Chlorogenic acid
Cell-cycle arrest	↓ cyclin D, E ↓ CDK 1, 2, 4 ↓ PCNA	EGCG
	↑ cyclin E ↓ cyclin A, B1	Ellagic acid
Apoptosis	↑ ROS in cancer cells ↑ caspase-3, -7, -8 ↑ cytochrome c ↑ Blc-2, Blc-X_L_	EGCG
	↑ caspase-3, -7, -9 ↑ cytochrome c ↑ PARP cleavage	Quercetin
Anti-proliferation/Anti-survival	↓ GFR/Ras/MAPK & PI3K/Akt ↓ c-fos ↓ erg-1 ↓ PI3K ↓ ERK ↓ Akt phosphorylation ↓ NF-κΒ	EGCG
Anti-inflammatory action	↓ COX-1 ↓ COX-2	Gallic acid
	↓ TNF-α ↓ COX-2	EGCG
Anti-angiogenesis	↓ VEGF ↓ PDGF ↓ HIF-1α	EGCG, anthocyanin berry extracts
Metastasis inhibition	↓ MMP-9 ↓ mRNA stabilizing factor HuR	EGCG
Cell adhesion and movement inhibition	↓ MRLC phosphorylation ↓ Vimentin phosphorylation	EGCG

Abbreviations: Akt/PKB, Protein kinase B; CDK, Cyclins-dependent kinase; COX, Cyclooxygenase; CYP, Cytochrome P450; ERK, Extracellular regulated kinase; GFR, Growth factor receptors; GSH, Glutathione; HIF-1α, Hypoxia-inducible factor 1α; LDH, Lactate dehydrogenase; MAPK, Mitogen-activated protein kinase; MMP-9, Matrix metallopeptidase 9; MRLC, Myosin II regulatory light chain (protein); NF-κB, Nuclear factor—kappa (κ) B; NQO, NADPH quinone oxidoreductase; PARP, Poly-ADP ribose polymerase; PCNA, Proliferating cell nuclear antigen; PDGF, Platelete-derived growth factor; PI3K, Phosphatidylinositol-3-kinase; ROS, Reactive oxygen species; TNF-α, Tumor necrosis factor alpha (α); UDPGT, UDP-glucuronosyl transferase; VEGF, Vascular endothelial growth factorr. ↑ Up-vector indicates induction/increase. ↓ Down-vector indicates suppression/decrease. * Other berry-derived bioactive compounds may confer similar response.

## Supplementary Materials

The following are available online at www.mdpi.com/2076-3921/5/4/37/s1. Table S1. Berries with selected nutrient and phytochemical profiles expressed in values per 100 g of edible portion as determined by USDA.
