# Supplementary material for: Protective Role of Dietary Berries in Cancer
Source: Antioxidants (Basel). 2016 Oct 19;5(4):37. doi: 10.3390/antiox5040037 (PMC5187535; doi:10.3390/antiox5040037)
Supplement: Supplementary file 1 [file antioxidants-05-00037-s001.pdf]

Supplementary Materials: Protective Role of Dietary Berries in Cancer

Aleksandra S. Kristo, Dorothy Klimis-Zacas, Angelos K. Sikalidis

Table S1. Berries with selected nutrient and phytochemical profiles expressed in values per 100 g of edible portion as determined by United States Department of Agriculture (USDA).

Berry Type	Anthocyanidin (mg) *	Flavan-3-ols (mg) †	Flavonols (mg) ‡	Vitamin C (mg)	Vitamin E (mg)
Bilberry	430.91	4.13	◊	◊	◊
Black currant	272.44	1.17	12.69	181	1.00
Black raspberry	324.02	◊	◊	◊	◊
Blackberry	90.46	42.50	2.49	21	1.17
Blueberry	163.52	51.71	9.72	9.70	0.57
Chokeberry	437.22	◊	8.90	◊	◊
Cranberry	0.72	◊	6.91	◊	◊
Mulberries	◊	◊	2.47	36.40	0.87
Red raspberry	◊	◊	1.32	26.20	0.87
Strawberry	33.63	4.51	1.60	58.50	0.29

* Total anthocyanidins: cyanidin, delphinidin, peonidin, petunidin. † Total flavan-3-ols: (−)-epicatechin, (−)-epicatechin 3-gallate, (−)-epigallocatechin, (−)-epigallocatechin 3 gallate, (+)-catechin, (+)-gallocatechin. ‡ Total flavonols: kaempferol, myricetin, quercetin. ◊ Value is not provided by the USDA food composition database. NOTE: Values for vitamins C and E were included due to the anti-oxidant potential of those nutrients. Source of data: USDA food composition database; modified from Basu et al., 2010 [1,2].

References

1. Basu, A.; Rhone, M.; Lyons, T.J. Berries: emerging impact on cardiovascular health. *Nutr. Rev.* **2010**, *68*, 168–177.
2. United States Department of Agriculture. Available online: www.usda.gov (accessed on 15 June 2016).

© 2016 by the authors. Submitted for possible open access publication under the terms and conditions of the Creative Commons Attribution (CC-BY) license (http://creativecommons.org/licenses/by/4.0/).
